# Cognitive impairment assessed by static and dynamic changes of spontaneous brain activity during end stage renal disease patients on early hemodialysis

**DOI:** 10.3389/fneur.2025.1510321

**Published:** 2025-02-18

**Authors:** Yunfan Wu, Rujin Li, Guihua Jiang, Ning Yang, Mengchen Liu, Yanying Chen, Zichao Chen, Kanghui Yu, Yi Yin, Shoujun Xu, Bin Xia, Shandong Meng

**Affiliations:** ^1^Department of Medical Imaging, The Affiliated Guangdong Second Provincial General Hospital of Jinan University, Guangzhou, China; ^2^The Second School of Clinical Medicine, Southern Medical University, Guangzhou, China; ^3^Department of Medical Imaging, Guangdong Second Provincial General Hospital, School of Medicine, Jinan University, Guangzhou, China; ^4^Department of Radiology, Shenzhen Children’s Hospital, Shenzhen, China; ^5^Department of Medical Imaging, Guangdong Medical University, Zhanjiang, China; ^6^The Department of Renal Transplantation, The Affiliated Guangdong Second Provincial General Hospital of Jinan University, Guangzhou, China

**Keywords:** hemodialysis, cognitive impairment, depression, static, dynamic, regional homogeneity

## Abstract

**Background:**

Compared with the general population, patients with end-stage renal disease (ESRD) undergoing maintenance hemodialysis (ESHD) exhibit a higher incidence of cognitive impairment. Early identification of cognitive impairment in these patients is crucial for reducing disability and mortality rates. Examining the characteristics of static and dynamic regional spontaneous activities in ESHD cases may provide insights into neuropathological damage in these patients.

**Methods:**

Resting-state functional magnetic resonance images were acquired from 40 patients with early ESHD (3 or 4 times/week for more than 30 days but less than 12 months) and 31 healthy matched controls. Group differences in regional static and dynamic regional homogeneity (ReHo) were identified, and correlations examined with clinical variables, including neuropsychological scale scores, while controlling for covariates. Receiving operating characteristic (ROC) curve analyses were conducted to assess the accuracy of ReHo abnormalities for predicting cognitive decline among early ESHD.

**Results:**

The ESHD group exhibited significantly reduced static and dynamic ReHo in the temporal and parietal lobes, including regions involved in basal ganglia–thalamus-cortex circuits, the default mode network, and ventral attentional network. Several static and dynamic ReHo abnormalities (including those in the right parietal and left middle temporal gyrus) were significantly correlated with neurocognitive scale scores. In addition, the dynamic ReHo value of the left superior temporal gyrus was positively correlated with depression scale scores. Comparing the ROC curve area revealed that numerous brain regions with altered ReHo can effectively distinguish between patients with ESHD and those without cognitive impairment.

**Conclusion:**

Our study found that spontaneous activity alterations located in the basal ganglia-thalamus-cortex circuit, default mode network, and ventral attentional network are associated with the severity of cognitive deficits and negative emotion in early ESHD patients. These findings provide further insight into the relationship between cognitive impairment and underlying neuropathophysiological mechanisms underlying the interplay between the kidneys and the nervous system in ESRD patients, and provide further possibilities for developing effective clinical intervention measures.

## Introduction

Chronic kidney disease (CKD) constitutes a major global public health issue (1). End-stage renal disease (ESRD) represents the final stage of CKD. The low glomerular filtration rate and the presence of proteinuria are both associated with the development of cognitive impairment and poor cognitive function (2). Compared to patients in other stages of CKD, ESRD patients have a higher risk of developing cognitive impairment. Previous studies (3, 4) have also shown that patients with ESRD undergoing maintenance hemodialysis (ESHD) exhibit markedly elevated rates of cognitive dysfunction and dementia. Rates of cognitive dysfunction as high as 49.1% have been reported among this clinical group (3). Moreover, patients with ESHD have cognitive impairment face greater mortality risk, especially elderly patients with ESHD who carrying dementia is associated with a twofold higher mortality risk compared to patients with ESHD who have not maintained hemodialysis (4). Conversely, the most common pathogenesis of cognitive impairments related to ESHD have been attributed to worsening brain edema, fluctuations in cerebral blood flow, changes in vascular permeability, and the presence of various uremic toxins (5). However, specific neuropathological mechanisms underlying the crosstalk between the kidneys and the nervous system remain elusive (6). Effectively distinguishing between patients with early ESHD with and those without cognitive impairment is crucial for exploring neuropathological mechanisms underlying the crosstalk between the kidneys and the nervous system, developing targeted prevention strategies, and mitigating disability and mortality rates in this population.

Resting-state functional magnetic resonance imaging (rs-fMRI) (7) based on blood oxygen level-dependent signals is a popular noninvasive modality for studying the neurobiological mechanisms underlying neuropsychiatric disorders, and several recent studies (8–10) have adopted rs-fMRI to identify abnormalities in regional neural activity associated with cognitive impairments among patients with ESHD. Static regional homogeneity (sReHo) is a rs-fMRI-derived metric that reflects the consistency of spontaneous neural activity between a given voxel and its neighboring voxels as reflected by Kendall’s coefficient of conformity (KCC) (11). This metric has been widely used in investigating abnormal spontaneous neural activity in various neuropsychiatric disorders associated with cognitive impairment, including mild cognitive impairment (12), vascular cognitive impairment (13), hypertension (14), and schizophrenia (15). Xue et al. reported reduced regional sReHo in multiple areas of bilateral frontal, parietal, and temporal lobes among patients with ESRD and those without neurocognitive dysfunction regardless of hemodialysis history (16). In patients with ESHD, Chen et al. observed that sReHo values decreased in multiple cortical regions, including components of the default mode network such as the bilateral precuneus, posterior cingulate cortex, medial prefrontal cortex, inferior parietal lobe, right angular gyrus, right postcentral gyrus, bilateral superior temporal gyrus, and right supramarginal gyrus (17). Yu et al. found abnormal sReHo and amplitude of low-frequency fluctuation values in the basal ganglia, cerebellum, and hippocampus of patients with CKD with and without dialysis (18). These studies have demonstrated that sReHo methods effectively identify abnormal brain regions associated with cognitive decline in in patients with CKD/ESRD regardless of dialysis status.

However, multiple studies have demonstrated that the brain dynamically integrates or adjusts its response to stimuli across various time scales (19, 20). This capability is crucial for understanding the underlying neural mechanisms of behavior and cognitive impairment in individuals with mental illness and neurological disorders (19). Many recent studies have focused on the relationship between altered brain dynamic functional connectivity (dFC) and cognitive deficits in ESRD patients, giving a fresh research perspective. For example, Cao et al. found that dFC alterations in the triple network model (between the default mode network, the salient network, and the central executive network) may be a pathophysiological mechanism for cognitive impairment in ESRD patients on hemodialysis (21); Li et al. analyzed dFC of whole brain networks in ESRD patients, and found that the impaired functional flexibility of network connectivity and the disruption of state-specific FCs may be the basis of their cognitive deficits (22). Similarly, compared to sReho, dynamic Reho can captures multiple snapshots of brain homeostasis at specific time points, wiftly captures the spatiotemporal dynamic characteristics of the brain, offers detailed neural information about time-varying functional networks and may offer greater sensitivity in detecting temporal dimension information in regional neural activity synchronization. Consequently, it facilitates a deeper exploration of how different brain regions dynamically coordinate during various cognitive tasks (23, 24). Dynamic ReHo has been extensively applied in various studying various cognitive impairments in neuropsychiatric and degenerative disorders, such as mild cognitive impairment (25), Alzheimer’s disease (26), obstructive sleep apnea (27), and type 2 diabetes (28). For instance, Chun et al.’s study on Alzheimer’s disease revealed abnormal dynamic ReHo in the right frontal gyrus and right cingulate gyrus, correlating with decreased cognitive abilities. Similarly, Yang et al.’s research on patients with mild cognitive impairment, comparing those with and without depressive symptoms, identified significant differences in dynamic ReHo in the frontal gyrus, temporal gyrus, and parietal lobe. These findings suggest that dynamic whole-brain functional activity can potentially serve as early biomarkers for detecting cognitive deficits and emotional issues in individuals with mild cognitive impairment. However, to the best of our knowledge, no study has identified brain regions of cognitive impairment or emotion symptoms through dReHo analysis among early ESHD cases.

In summary, integrating static ReHo (sReHo) and dynamic ReHo (dReHo) indicators can offer a more comprehensive understanding of the neuropathological changes in ESHD. As far as we know, we firstly employed sReHo and dReHo to investigate the temporal variability of voxel-wise brain activity and compared regional spontaneous activity changes between patients with early ESHD and matched controls. We hypothesized that early ESHD patients would exhibit abnormal spontaneous brain activity compared with those matched controls, particularly those implicated in processing cognitive impairment and emotional symptoms.

## Materials and methods

### Subjects

This prospective study was approved by the Affiliated Guangdong Second Provincial General Hospital of Jinan University Human Research Ethics Committee, and all participants provided written informed consent. From 2019 to 2021, we recruited 40 patients with early ESHD, defined as ESRD patients receiving hemodialysis 3 or 4 times per week for more than 30 days but less than 12 months as a previous study (29), and 31 age-, sex-, and education level-matched healthy control (HC) subjects. All of patients with ESRD were diagnosed as stage 5 of CKD based on the K/DOQI classification, with the glomerular filtration rate being almost completely reduced (<15 mL/min/1.73 m^2^). Eligibility criteria for both groups were age 20–65 years and right-handed. Exclusion criteria for both groups were drug abuse or alcohol dependence history, known severe neurological disorders (e.g., stroke, epilepsy, intracranial malignancy), psychiatric disorders including schizophrenia, anxiety or depression (defined by Self-Rating Depression Scale scores <50 and Self-Rating Anxiety Scale scores <53), severe head trauma, obvious brain lesions (subjects with stroke, WMH Fazekas grade > 2, tumor or traumatic brain injury were excluded from both the ESHD group and the HC group), and contraindications or intolerance to magnetic resonance (MR) imaging. Cognitive impairment in ESHD cases was diagnosed by two neurologists with 15 years of experience. All clinical data were collected from patients’ electronic medical records.

### Neuropsychological testing

Patients underwent the following multiple neuropsychological tests (30, 31) before MR data acquisition and before 2 h undergoing hemodialysis: the Folstein version of the Mini-Mental State Examination (MMSE), Montreal Cognitive Assessment (MoCA), digital connections test types A (NCT-A), line-tracing test (LTT), serial dot test (SDT), digit symbol test (DST), Self-rating Depression Scale (SDS), and Self-rating Anxiety Scale (SAS). The MoCA and MMSE are two of the most widely used screening tests for mild cognitive decline while the SDS and SAS are common screening tools for depression and anxiety, respectively. The NCT-A was used to assess visual perception and motor reaction time, whereas the NCT-B was used to assess visual perception, working memory, attention, and executive control. The LTT was used for assessment of visual discrimination and attention, the SDT for spatial ability, reaction speed, and fine motor skills assessment, and the DST for assessment of short-term memory, reaction speed, and accuracy of attention accuracy. Trained psychometricians administered these neuropsychological tests. Consistent with previous studies (32, 33), individuals with SAS score ≥ 50 were classified as experiencing anxiety, whereas those with SDS score ≥ 53 were classified as having depression.

### Laboratory tests

All patients with ESHD also received comprehensive parameters of laboratory examinations to measure blood hemoglobin as well as serum urea nitrogen, creatinine, calcium, and potassium levels within 24 h of fMRI examination. Blood laboratory tests were not conducted in the HC group.

### MR data acquisition

MR data were obtained using a Philips Ingenia 3.0 T MR scanner system with a 32-channel phased-array head coil at the Department of Medical Imaging, Guangdong Second Provincial General Hospital. Each subject was in a supine with eyes closed and the head snugly restricted by a belt and foam pads as described previously (34). Single-shot echo-planar imaging (EPI) was applied for each subject using the following two sequences: axial slices, 33; repetition time (TR), 2000 ms; echo time (TE), 30 ms; flip angle (FA), 90°; slice thickness, 3.5 mm with no gap; matrix, 64 × 64; field of view (FOV), 230 mm × 230 mm^2^. A total of 240 volumes were obtained per participant. Further, individual three-dimensional T1-weighted images (T1WI) were acquired by an EPI sequence (160 sagittal slices; TR, 25 ms; TE, 4.1 ms; FA, 30°; slice thickness, 1.0 mm with no gap; FOV, 230 × 230 mm^2^; and matrix, 230 mm × 230 mm, total volume of 240). Each rs-fMRI scan lasted 8 min.

All subjects were scanned T2 fluid-attenuated inversion recovery (FLAIR) sequence to exclude obvious brain lesions. MR images were examined by two physicians with ≥15 years of experience to exclude image quality problems such as obvious artifacts.

### MR imaging analysis

The rs-fMRI datasets were preprocessed and analyzed using the DPARSFA 5.3 Advanced Edition plugin for DPABI 6.2_220915^1^ (35). Preprocessing included the following steps: (1) removal of the first 10 images to eliminate the influence of unstable magnetization, (2) correcting the remaining 230 images for temporal differences (slice timing realignment), (3) removal of scans with head motion >1.5 mm in translation or 1.5° in rotation (however, no subject was eliminated because head movements did not exceed these thresholds), (4) normalization of all functional images, registration to the standard Montreal Neurological Institute template using the DARTEL method, and resampling at a resolution of 3 × 3 × 3 mm^3^, (5) linear detrending processing, (6) regressing out the Friston-24 head motion parameters, white matter signal, and cerebrospinal fluid signal using nuisance covariate regression (there were no significant differences in the head movement parameters between the two groups), and (7) band-pass filtering at 0.01–0.08 Hz. During all preprocessing steps, two radiologists with ≥15 years of experience checked the images to ensure segmentation quality and correct registration.

### Computation of static and dynamic regional homogeneity

Regional sReHo was computed from the KCC of each voxel using DPABI software as reported in previous studies (11). The sReHo map of each subject was then constructed and transformed into a standardized z-score map.

Regional dReHo was calculated using temporal dynamic analysis toolkits based on DPABI V6.2 (36). Previous studies have shown that the setting of the dynamic window width should try to strike a balance between large enough and small enough so that not only the lowest frequencies in the signal can be analyzed, but also potential transients can be detected. Therefore, based on previous studies, we used a sliding window of medium length of 60 s (30 repetition times [TRs]) and a shift step of 2 s (1 TR) to calculate the temporal variability of dReHo (26, 27, 37). For each sliding window, an ReHo graph was constructed to estimate dReHo. Mean dReHo of the whole brain was calculated to normalize each voxel of the ReHo map by z-transformation. Finally, sReHo and dReHo maps were smoothed using an 8-mm full-width at half maximum Gaussian kernel prior to statistical analyses.

### Statistical analysis

Group differences in demographic and clinical variables were evaluated using SPSS 20.0 software (SPSS Inc., Chicago, USA). All continuous datasets were first assessed for normality using the Shapiro-Wilk test. Two-sample t-tests assessed intergroup differences in age, education, neuropsychological test scores and clinical laboratory indicators. Sex ratio was compared between groups using the chi-square test.

Group differences in imaging parameters and associations between these parameters and clinical metrics including cognitive test scores were evaluated using Matlab 2016 (Math Works, USA). DPABI V6.2 was used to compare sReHo and dReHo between ESHD and HC groups. Mean z-transformed ReHo maps were compared between groups by two-sample t-test analysis. And then, multiple comparisons of the differential brain region results between the two groups were corrected using the Gaussian Random Field (GRF) method with voxel-level *p* < 0.001 and cluster-level *p* < 0.05; all clusters included at least 20 voxels. Mean sReHo and dReHo values for all voxels differing significantly were extracted separately using the resting-state fMRI Data Analysis Toolkit (REST; http://resting-fmri.sourceforge.net). Voxel clusters differing significantly in mean sReHo or dReHo are displayed in MNI coordinates. Finally, associations between mean sReHo and dReHo z-values of patients differing significantly from healthy controls and clinical variables (cognitive and emotional scales scores, parameters of laboratory examinations, the duration of hemodialysis) were evaluated by partial correlation tests with age, sex, and years of education as covariates (Bonferroni correction). A *p* < 0.05 was considered statistically significant for all correlation coefficients.

Additionally, receiving operating characteristic (ROC) curves were constructed to assess the capacities of regional sReHo and dReHo values to distinguish between patients with ESHD possessing and lacking cognitive impairments. The area under each ROC curve (AUC) was calculated and expressed as AUC ± standard deviation (SD). Cutoff values were selected based on the highest Youden index, calculated as [1 − (1 − sensitivity) (1 − specificity)]. Probability (P) values of <0.05 were considered statistically significant. Data are presented as mean ± SDs when normally distributed or medians with interquartile ranges when non-normally distributed.

## Results

### Demographic and clinical features

Forty patients with ESHD (30 days < dialysis duration<12 months, 3–4 times a week) were included. 18 patients had diabetes and 38 patients had hypertension. There was no significant difference in age, sex, education (all *p* > 0.05). In contrast, the NCT-A/B and DST, SDT, LTT, SDS, and SAS scores of the ESHD group were significantly higher than that of the HC group (*p* < 0.05), and the MoCA and MMSE scores of the ESHD group were significantly lower than that of the HC group (p < 0.05) (Table 1).

**Table 1:** Demographics and clinical characteristics of all participants.

Characteristic	ESRD participants on during early-stage hemodialysis(ESHD group; *n* = 40)	HC participants (*n* = 31)	*p* value
Age (years)	44.27 ± 11.18	44.13 ± 10.63	0.528
Sex (male/female)	21/19	18/13	0.810
Duration of education (yeas)	10.95 ± 5.43	9.71 ± 4.10	0.852
MMSE (score)	26.75 ± 2,79	28.45 ± 1.75	<0.001
MoCA (score)	24.73 ± 3.39	27.45 ± 2.20	0.004
NCT-A (s)	66.45 ± 37.42	45.90 ± 10.44	<0.001
NCT-B (s)	77.45 ± 67.81	77.16 ± 29.57	0.004
DST (score)	39.88 ± 21.49	51.39 ± 5.94	<0.001
SDT (s)	70.85 ± 42.10	44.13 ± 10.73	0.027
LTT (s)	60.00 ± 26.46	44.19 ± 8.78	<0.001
SDS (score)	33.78 ± 10.29	18.55 ± 4.64	<0.001
SAS (score)	32.00 ± 8.35	17.74 ± 4.88	0.080
Hemoglobin (g/l)	94.40 ± 19.18	/	/
serum urea nitrogen (mmol/L)	24.67 ± 9.17	/	/
serum creatinine (μmol/L)	987.98 ± 359.36	/	/
serum Kalium (mmol/l)	4.76 ± 0.68	/	/
serum Calcium (mmol/l)	2.29 ± 0.76	/	/

### Group differences in sReHo

**Figure 1:**
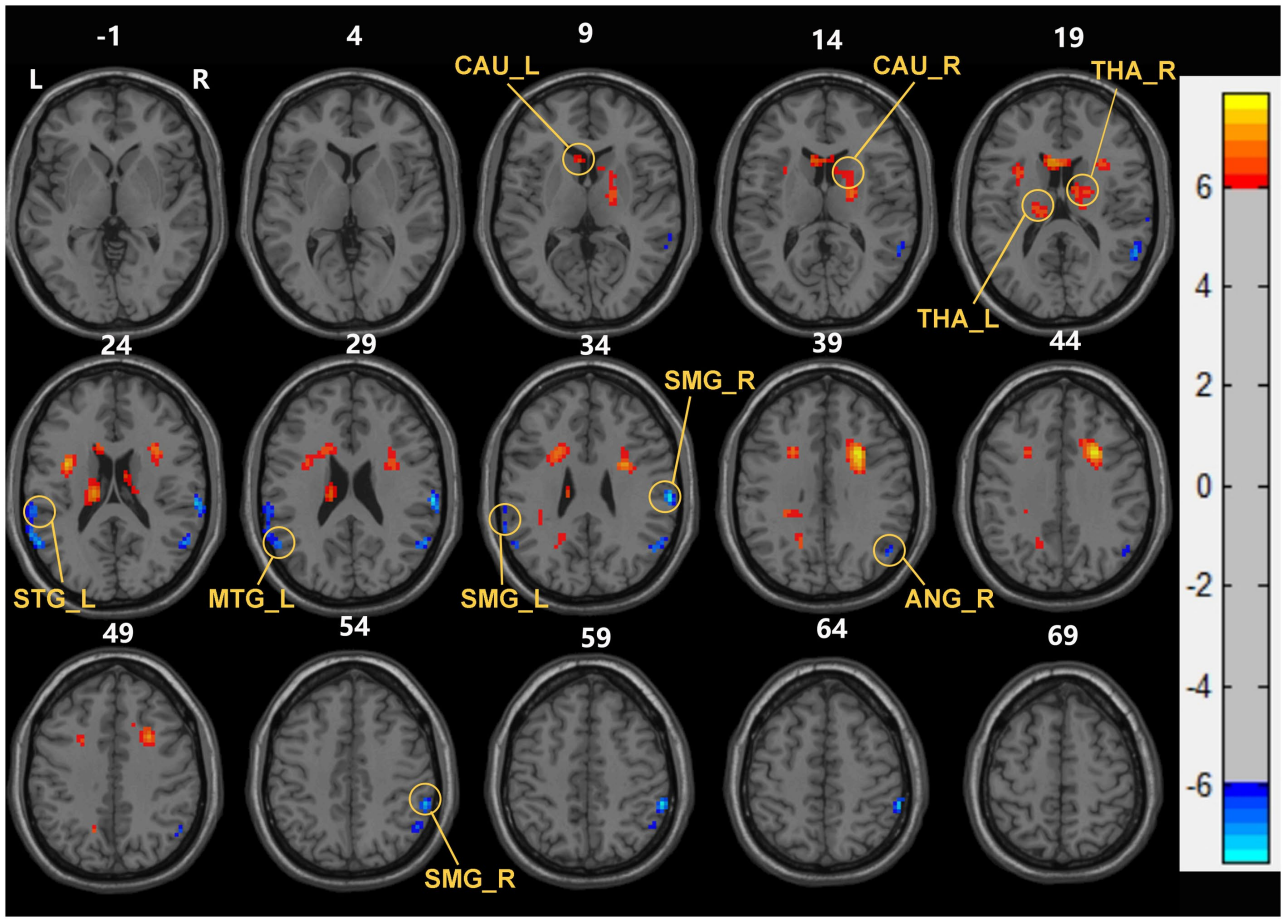
Brain areas with significant sReHo differences between early end stage renal disease patients undergoing maintenance hemodialysis (ESHD) groups and healthy controls (HC) groups. Regions in red-yellow are brain areas where sReHo was significantly increased in ESHD groups compared to HC groups. Regions in blue-green are brain areas where sReHo was significantly decreased in ESHD groups compared to HC groups. The results were multiple compared at the voxel-level (two-tailed voxel-level: *p* < 0.001, Gaussian Random Field correction, with a cluster size >20 voxels, cluster-level: *p* < 0.05).

Compared with the HC group, ESHD patient during the first year exhibited significantly increased sReHo in bilateral caudate nucleus (CAU), bilateral thalamus (THA), and decreased sReHo in the right angular gyrus (ANG), bilateral supramarginal gyrus (SMG), left superior temporal gyrus (STG) and left middle temporal gyrus (MTG) (Table 2; Figure 1).

**Table 2:** Brain regions with different sReHo and dReHo between ESHD and HCs group.

Indices	Brain region	MNI coordinate	Voxels	Peak intensity	Cohen’s f^2^
sReHo	ANG_R	50, −61, 39	146	−8.293	0.638
	SMG_R	63, −21, 27	62	−7.527	0.849
	SMG_L	−65, −27, 29	56	−5.760	0.513
	STG_L	−66, −31, 17	39	−5.849	0.494
	MTG_L	−55, −58, 17	33	−7.181	0.567
	CAU_R	16, 10, 10	52	6.001	0.493
	CAU_L	−10, 19, 10	21	6.199	0.579
	THA_R	20, −18, 4	48	5.932	0.456
	THA_L	−18, −32, 8	25	5.470	0.447
dReHo	ANG_R	48, −66, 39	146	−8.293	0.300
	SMG_R	63, −20, 25	130	−6.994	0.260
	IPL_R	57, −42, 51	89	−8.405	0.341
	PoCG_R	63, −6, 21	39	−6.162	0.205
	ANG_L	57, −42, 51	81	−8.301	0.341
	STG_L	−63, −33, 20	37	−6.896	0.285
	MTG_L	−57, −57, 21	23	−6.596	0.288
	MFG_L	−41, 26, 35	28	−5.381	0.112

ANG, angular gyrus; SMG, supramarginal gyrus; STG, superior temporal gyrus; MTG, middle temporal gyrus; MFG, middle frontal gyrus; IPL, Inferior parieta gyrus; PoCG, postcentral gyrus; CAU, caudate nucleus; THA, thalamus. L; left; R; right. ESHD, end stage renal disease patients undergoing maintenance hemodialysis; HC, healthy control.

### Group differences in dReHo

**Figure 2:**
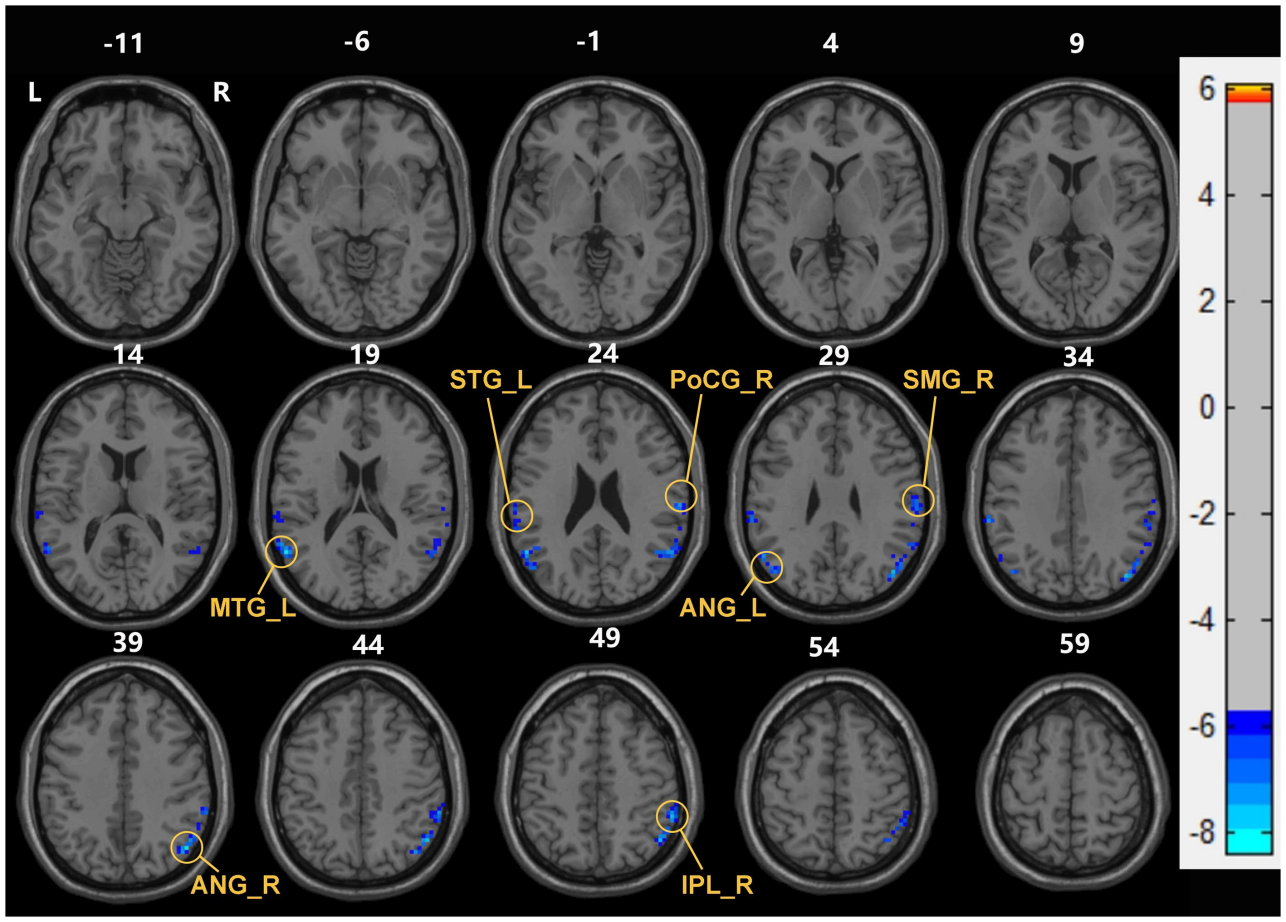
Brain areas with significant dReHo differences between early end stage renal disease patients undergoing maintenance hemodialysis (ESHD) groups and healthy controls (HC) groups. Regions in blue-green are brain areas where dReHo was significantly decreased in ESHD groups compared to HC groups. The results were multiple compared at the voxel-level (two-tailed voxel-level: *p* < 0.001, Gaussian Random Field correction, with a cluster size >20 voxels, cluster-level: *p* < 0.05).

Compared with the HC group, decreased dReHo in multiple brain regions including bilateral ANG, right SMG, right postcentral gyrus (PoCG), right inferior parietal gyrus (IPL), left STG, left MTG, and left middle frontal gyrus (MFG) (Table 2; Figure 2).

### Correlation analysis

**Figure 3:**
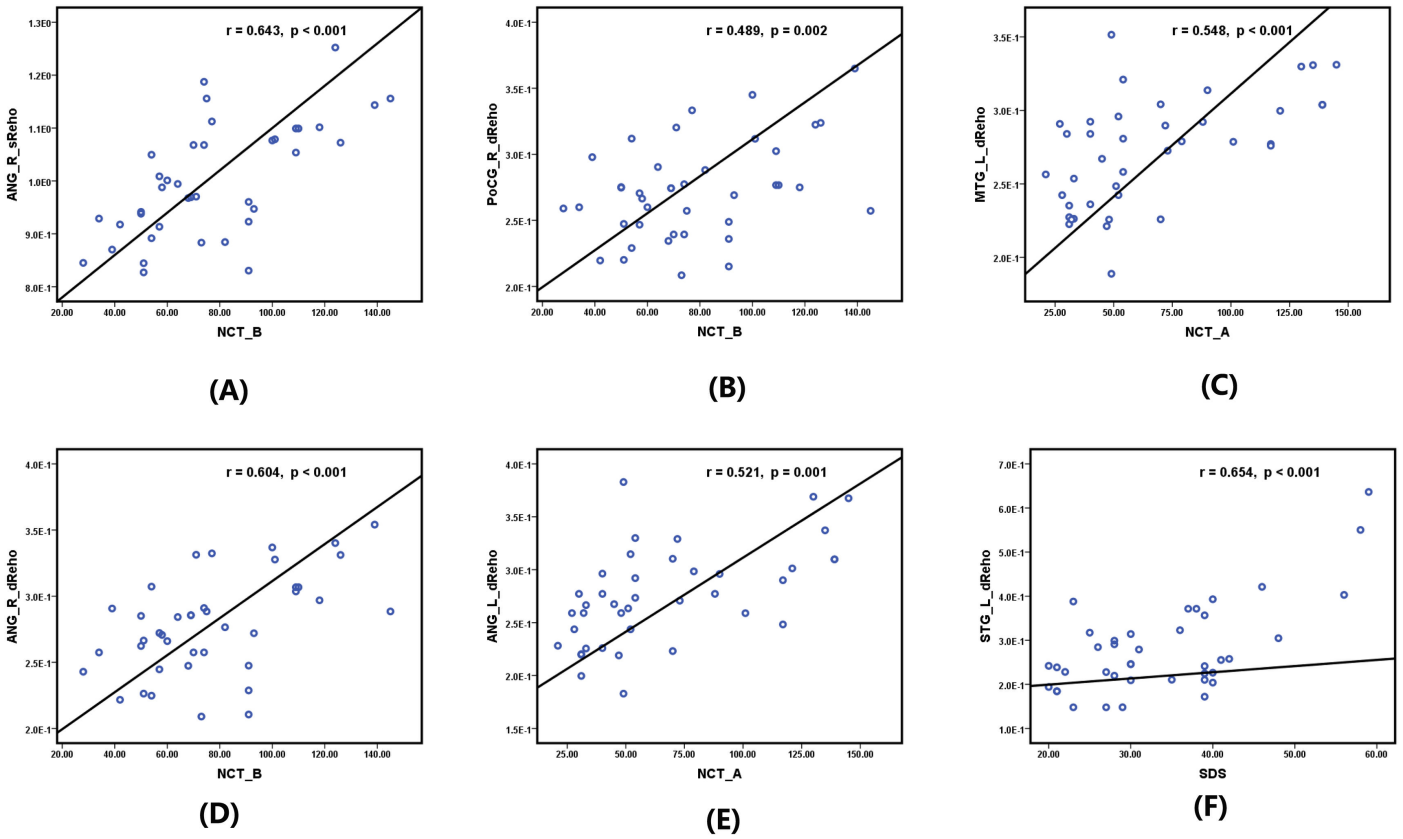
Significantly positive correlation between sReHo/dReHo values and neurocognitive and emotion scale scores in hemodialysis patients in the first year: **(A)** between the sReHo value of right ANG and the NCT-B score. **(B)** Between the sReHo value of the right PoCG and the NCT-B score. **(C)** Between the dReHo value of the left MTG and the NCT-A score. **(D)** Between the dReHo value of the right ANG and the NCT-B score. **(E)** Between the dReHo value of the left ANG and the NCT-A score. **(F)** Between the dReHo values of the left STG and the SDS score.

**Figure 4:**
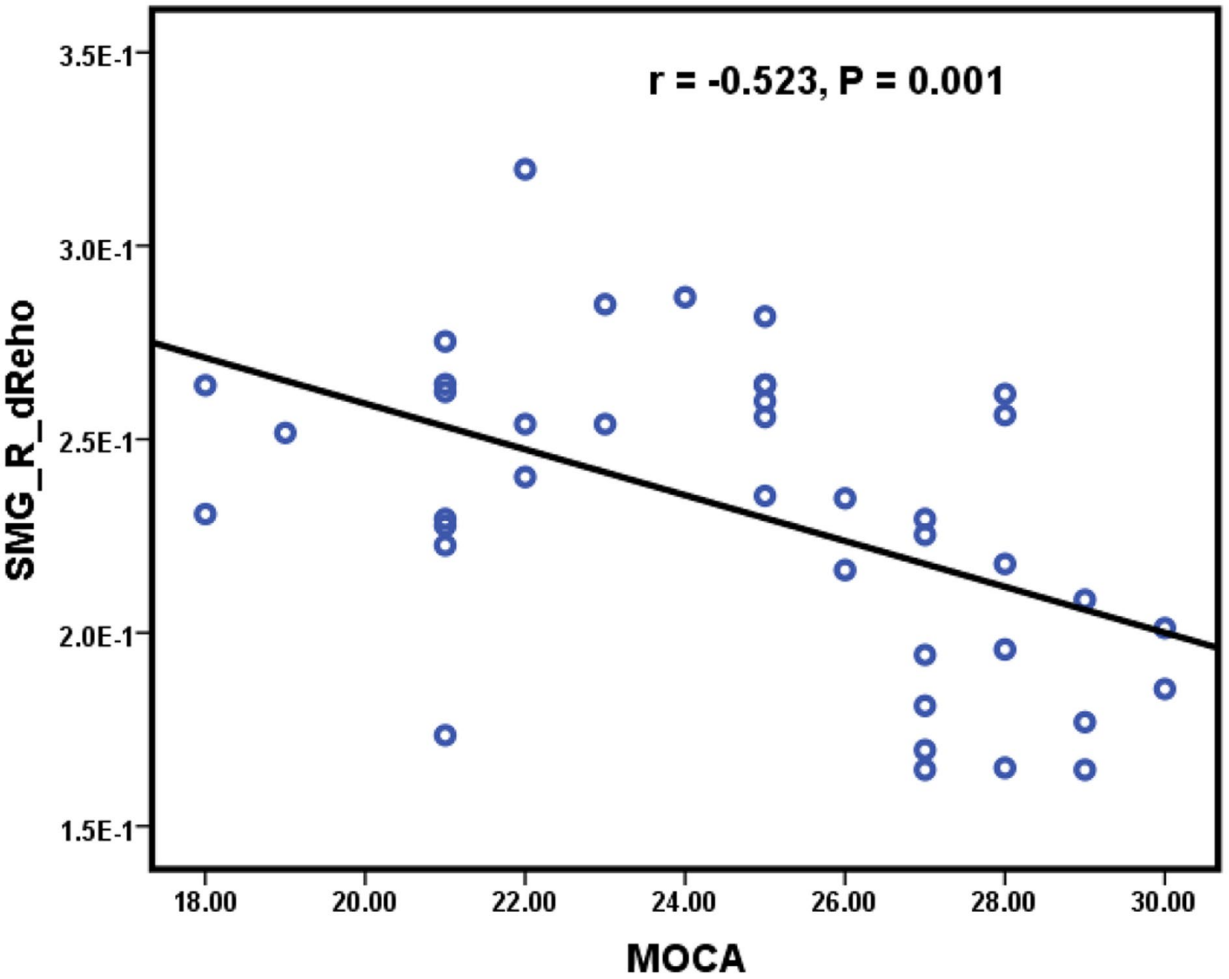
Significantly negative correlations were found between the dReHo value of the right SMG and the MoCA score.

As shown in Figure 3, significant positive correlations were found between the sReHo value of the right ANG and the NCT-B score (r = 0.643, *p* < 0.001) (Figure 3A), the dReHo value of the right PoCG and the NCT-B score (r = 0.489, *p* = 0.002) (Figure 3B), the dReHo value of the right ANG and the NCT-B score (r = 0.604, *p* < 0.001) (Figure 3D), the dReHo value of the left ANG and the NCT-A score (r = 0.521, *p* = 0.001) (Figure 3E), the dReHo value of the left MTG and the NCT-A score (r = 0.548, *p* < 0.001) (Figure 3C), and the dReHo values of the left STG and the SDS score (r = 0.654, *p* < 0.001) (Figure 3F). In addition, negative correlations were found between the dReHo value of the right SMG and the MoCA score (r = −0.523, *p* = 0.001) (Figure 4). Correlations between blood indices (serum urea nitrogen, creatinine, calcium, and potassium levels) and sReHo and dReHo were not observed in our study.

**Figure 5:**
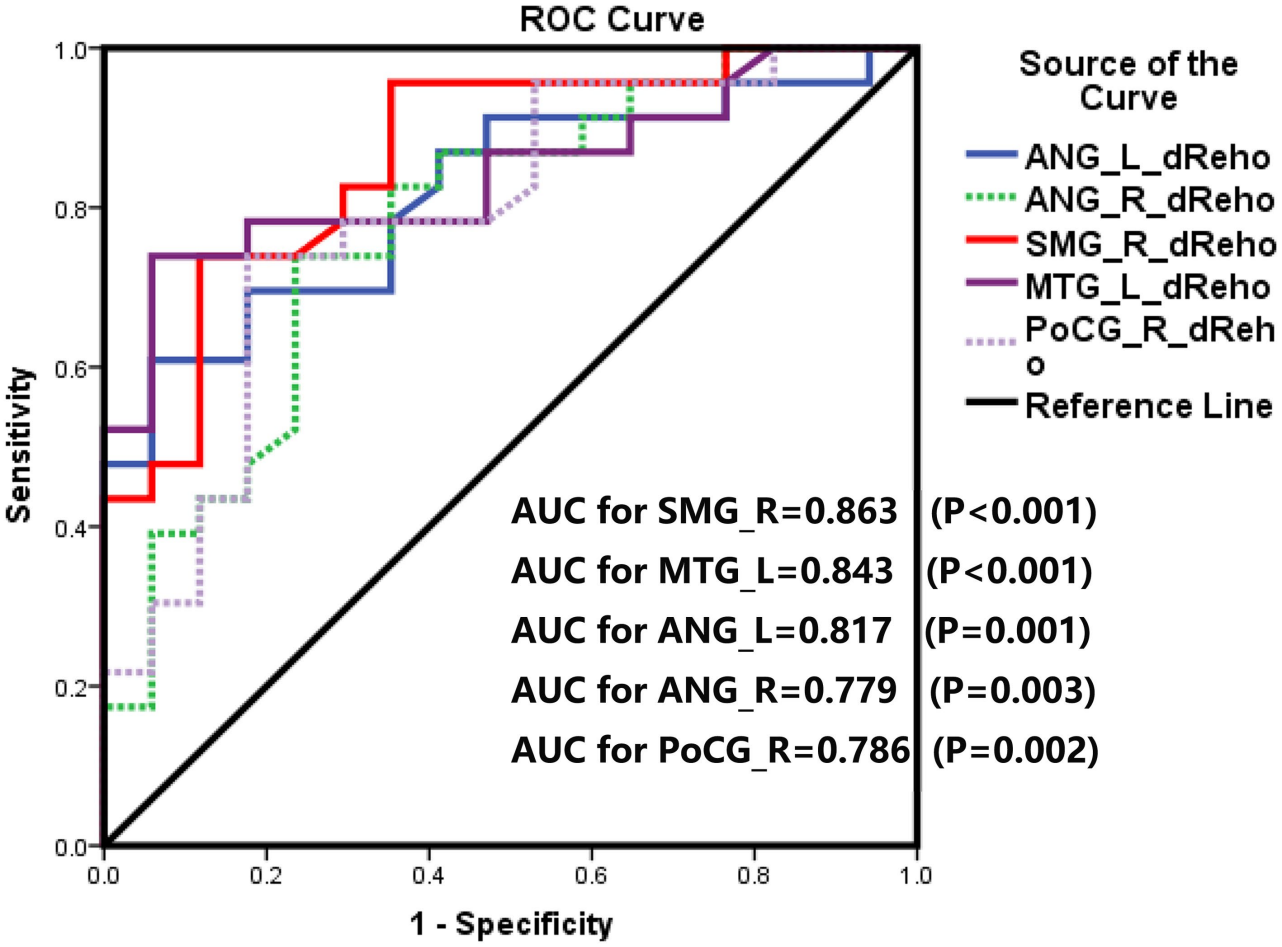
Receiver operator characteristic (ROC) curves for dynamic regional homogeneity values of the right supramarginal gyrus (SMG), left middle temporal gyrus (MTG), bilateral angular gyrus (ANG), and right postcentral gyrus (PoCG) to distinguishing between with and no-with concomitant cognitive in hemodialysis patients. AUC: area under the curve.

As shown in Figure 5 and Table 3, the AUCs for many alternative dReHo regions suggested that all can accurately identify patients with cognitive impairment and those without cognitive impairment during early hemodialysis. The AUCs for dReHo values were as follows: the right SMG (0.863, *p* < 0.001, sensitivity 73.9%, specificity 88.2%), left MTG (0.843, *p* < 0.001, sensitivity 73.9%, specificity 94.1%), left ANG (0.817, *p* = 0.001, sensitivity 60.9%, specificity 94.1%), right ANG (0.779, *p* = 0.003, sensitivity 73.9%, specificity 76.5%), and right PoCG (0.786, *p* = 0.002, sensitivity 73.9%, specificity 82.4%), respectively.

**Table 3:** Diagnostic efficiency of these differentiating dynamic regional homogeneity regions.

Brain regions	AUC	Sensitivity	Specificity	Youden index	Cut-off
SMG_R	0.863	73.9%	88.2%	0.62	2.35
MTG_L	0.843	73.9%	94.1%	0.68	2.80
ANG_L	0.817	60.9%	94.1%	0.55	2.91
ANG_R	0.779	73.9%	76.5%	0.50	2.74
PoCG_R	0.786	73.9%	82.4%	0.56	2.91

SMG, supramarginal gyrus; MTG, middle temporal gyrus; ANG, angular gyrus; PoCG, postcentral gyrus; L, left; R, right.

## Discussion

To our knowledge, this study represents the first comprehensive investigation into spontaneous neural brain activity in early ESHD patients with cognitive impairment, integrating both dReHo and sReHo measures. In this study, whole brain sReHo and dReHo maps were constructed using resting-state fMRI datasets to investigate regional spontaneous activity patterns in patients with early ESHD compared to matched healthy controls. The findings revealed significant alterations in both static and dynamic ReHo patterns in several brain regions. Specifically, patients with ESHD exhibited reduced sReHo and dReHo in the parietal and temporal lobes, increased sReHo in bilateral THA and CAU, and lower dReHo values in the MFG compared to controls. Furthermore, sReHo and dReHo abnormalities in several brain regions (right ANG, right SMG, right PoCG, and left MTG) were significantly correlated with neurocognitive scale scores in patients with ESHD. In addition, the dReHo value of the left STG was positively correlated with SDS score for depression. Importantly, ROC curve analysis showed that many of these regional abnormalities in sReHo and dReHo accurately distinguished patients with ESHD and those without cognitive decline.

Many studies have shown that there is an interaction between renal impairment and changes in neurological activity, which is called “kidney-brain axis” (38). Vascular and blood flow factors are now recognized as the main pathogenesis of cognitive impairment in CKD. The kidney and the brain have anatomically similar strain vessels. When there are some factors that are prone to damage the blood vessels, such as hypertension, diabetes, and hyperlipidemia, the renal and cerebral strain vessels will be damaged at the same time, and then show parallel renal and cerebral function impairment (39). On the other hand, the accumulation of various uremic toxins due to renal impairment also impairs neurological function by damaging the blood–brain barrier, inducing neuroinflammation, promoting apoptosis, disrupting brain neurotransmitters, and disrupting dopamine metabolism, among other pathways (40). ReHo metrics reflects the consistency of spontaneous neural activity between a given voxel and its neighboring voxels, and therefore neurological impairment through the “kidney-brain axis” is likely to affect ReHo (11). Our results showed that there were multiple brain regions with abnormal measurements of both sReHo and dReHo, which may be a reflection of the above mechanism.

Regions within the parietal cortex exhibiting reduced sReHo or dReHo included the bilateral ANG, right SMG, right PoCG, and right IPL. The parietal cortex is known for supporting a wide array of higher-order cognitive functions such as spatial representation, multimodal integration, attentional control, numeric judgment, motor planning, and working memory among other higher-order cognitive functions (41, 42). The ANG receives somatosensory input from more anterior parts of the parietal lobe, auditory input from the temporal lobe, and visual input from the occipital lobe (43), and functions in language and reading comprehension, mathematical, spatial, and social cognition, and episodic and semantic memory (43, 44). Similarly, the SMG contributes to episodic memory encoding, short-term auditory memory, and memory processing (45–47). The IPL supports spatial representation, multimodal integration, attentional control, numeric judgment, motor planning, working memory, and visual spatial attention (42, 48). Early hemodialysis patients frequently exhibit impairments in visual processing, reasoning, planning, short-term memory, and verbal processing, indicating broad parietal lobe dysfunction (29). Consistent with our findings, Chen et al. also found greater spontaneous brain activity in the ANG/IPL, PoCG, SMG, and precuneus among patients with ESHD as measured by sReHo (17) and amplitude of low-frequency fluctuation (ALFF) (8). In our study, we found significant correlations between sReHo or dReHo values in parietal lobe regions including the bilateral ANG, right SMG, and right PoCG with neurocognitive scale scores (NCT-A, NCT-B, and MoCA) in patients with early ESHD, highlighting the association between altered static and dynamic ReHo and cognitive impairment across multiple parietal lobe regions. On the other hand, the PoCG forms the anterior border of the inferior parietal cortex and contribute to complex linguistic and somatosensory processing. PoCG activity may lead to pervasive neurocognitive deficits. Moreover, Mastria and colleagues found that abnormal functional connections in the PoCG were associated with somatosensory abnormalities, leading to abnormal finger fine motor and tactile stimulation (49). Therefore, our speculation suggests that the positive correlation observed between abnormal regional homogeneity (dReHo) in the right PoCG and scores on the Neuropsychological Cognitive Test Battery (NCT-B) may indicate impairments in fine finger motor skills and somatosensory processing. Furthermore, our study demonstrated that the ROC curves constructed for the bilateral ANG and right SMG distinguished patients with early ESHD and those without cognitive impairment with good diagnostic efficiency. These finds strongly implicate widespread parietal cortex dysfunction as reflected by aberrant spontaneous activity in the cognitive impairments of patients with early ESHD.

We also found abnormal spontaneous activity of the STG and MTG in patients with early ESHD. First, the STG and MTG together with parietal lobe structures (SMG and ANG) form the auditory speech center known as Wernicke’s area (50). Lesions in this region lead to various forms of sensory aphasia, such as the inability to read (alexia), write (agraphia), compute (acalculia), and name objects (anomia) (51). Second, the temporoparietal junction and inferior parietal lobe are also part of the default mode network (DMN), mediating internal reflection, and are connected to the ventral attention network and executive control network. Lesions within these pathways are associated with deficits in language recognition, social cognition, situational memory retrieval, and attention reorientation (52, 53). Liao et al. found alterations in the dynamic intrinsic brain activity of the DMN were observed in individuals with AD, as evidenced by disrupted functional connectivity patterns compared to healthy controls (26). Fu et al. also observed significant alterations in the dynamics of brain spontaneous activity and functional networks, particularly affecting the DMN, in patients with type 2 diabetes who exhibited cognitive impairment compared to those without cognitive deficits (28). Our findings suggest that the aberrant dynamics within the DMN may underlie cognitive impairments observed in ESHD, reflecting compromised neuronal communication integrity crucial for higher-order cognitive processes. We further speculate that the abnormal ReHo values of STG and MTG may be related to the impaired emotional memory, executive function, and sensory language abilities observed in many patients with early ESHD. While previous studies of patients undergoing hemodialysis have also found abnormal spontaneous activity in parietal or temporal components of the DMN (8, 9, 17, 54), the patterns reported here are not entirely consistent. We speculate that different analysis methods and patient selection may contribute to these differences. Previous studies have used static methods of analysis, whereas our study used dynamic methods of analysis; moreover, perhaps there was heterogeneity in the criteria for grouping patients between the previous studies and our study. These may have contributed to the differences in results. Nonetheless, the current study highlights the importance of DMN dysfunction in the cognitive deficits observed among patients with early ESHD.

Compared to the HC group, patients with ESHD also exhibited lower dReHo in the MFG and higher sReHo in bilateral THA and CAU. The MFG is critical for planning and decision-making, executing and controlling attention, and completing working memory tasks (55). Regions of the MFG and the junction between the frontal gyrus and temporoparietal cortex belong to the ventral attentional network, which can be disrupted by visual or tactile stimuli, leading to top-down attentional network regulation and errors in working memory, learning, reward judgment, or emotional judgment (48, 56). The caudate nucleus of the basal ganglia is involved in neural pathways regulating emotional, motivational, associative, and cognitive functions (57). Previous structural and functional neuroimaging studies have also reported abnormalities in activity within this region among patients undergoing hemodialysis. For example, a voxel-based morphometry study by Wang and colleagues found a correlation between reduced left caudate volume and lower MMSE scores (indicative of general cognitive impairment) in patients undergoing hemodialysis (10), while Zhang and coworkers reported reduced long- and short-range functional connectivity density in the caudate as well as bilateral frontal, bilateral parietal, and left temporal lobes of patients with ESRD (58). The THA is a critical node in multiple functional circuits that regulate memory, emotion, attention, and information processing (59). Ma et al. found that a decrease in the ratio of N-acetylaspartate to creatine (NAA/Cr) in the thalamus of patients undergoing hemodialysis was associated with abnormal brain function (60) while others have reported that the functional connectivity strengths between THA and multiple cortical networks (including default mode, executive, dorsal attention, saliency, motor, visual, and auditory networks) are associated with various cognitive abilities, including processing speed, selective attention, and cognitive flexibility (61). Jin et al. found significant abnormalities in FC among bilateral thalamus, bilateral caudate, and bilateral temporal gyrus (62), and suggested that reduced integration in the basal ganglia–thalamus–cortex circuit may underlie functional impairments in patients undergoing hemodialysis. In light of these previous findings (57, 62) and our current observations, we speculate that functional abnormalities within the basal ganglia–thalamus–cortex circuit and ventral attentional network contribute to attention, learning, and behavioral deficits among patients with ESHD.

Interestingly, significant positive correlations between dReHo values of the left STG and SDS score were observed. DMN is considered to be closely related to self-referential mental activity and emotional processing (63). As an important component of the DMN, the STG is not only involved in social cognition and language expression, but also plays an important role in affective processing, participating in important functions such as emotional attention and perception (64, 65). Abnormal activity of DMN structures including the STG can lead to poor self-referencing and rumination, such as depression (66). Many studies have also suggested that the STG is one of the key structures involved in emotional processing in neural networks (67), and that the middle and superior temporal cortex is associated with explicit attention to emotional information (68). Therefore, STG is also closely related to emotion regulation. Abnormalities in STG activity have also been reported in various psychiatric disorders (69, 70), including depression and anxiety (65, 71). Previous studies (6, 72) have shown higher prevalence rates of anxiety and depression in patients with CKD or those undergoing hemodialysis compared with the general population. Depression is particularly prevalent among patients undergoing maintenance hemodialysis (6, 54). An F-18 fluorodeoxyglucose positron emission tomography study conducted by Chen et al. revealed decreased glucose metabolism in the left STG, left MTG, right IPL, and left ANG of patients with prediabetic CKD accompanied by depressive symptoms (54). Therefore, in conjunction with our findings, we speculated that the reduced dReHo in the left STG may be correlated with the influence of negative emotions in ESHD cases.

The present study has several limitations. First, we cannot completely exclude the impact of negative emotions (anxiety and depression) on the research results. Although we excluded patients with ESHD involving anxiety or depression (defined by SDS scores <50 and SAS scores <53) at the time of enrollment, baseline SDS and SAS scores were still significantly higher than in those HC subjects, which may be related to our selection of patient types. Therefore, more rigorous experimental research is necessary to eliminate the influence of negative emotions in ESHD cases, such as analyzing SDS and SAS scores as covariates. Second, the sample size was relatively small, reducing statistical power. Some important associations may have been missed. Therefore, ensuring the accuracy of our results may necessitate a larger sample size or data from diverse medical settings, including early-stage non-dialysis ESRD patients, ESRD patients with a history of dialysis for 2 years or more, and patients in stages 2–4 of CKD. Meanwhile, in order to increase the reliability of the correlation analysis, in the future, we will try other statistical methods such as linear regression analysis with covariates, and analyze the correlation between age and brain function and cognitive ability using Generalized Additive Models (GAMs). These areas also represent our future research directions. Third, no causal inferences can be drawn due to the cross-sectional study design. Longitudinal studies are needed to determine whether the associations between regional ReHo abnormalities and cognitive impairments in hemodialysis strengthen with dialysis time. Forth, we cannot exclude effects of ultrafiltration rate, other dialysis parameters, primary disease complications (such as hypertension, hypotension, diabetes, transient ischemic attack, dysregulation of parathyroid or thyroid hormones, depression, anxiety, and genetic anomalies) and dialysis timing (pre- or post-dialysis fMRI scans) on regional ReHo and cognitive test performance.

## Conclusion

Patients with ESRD receiving hemodialysis for less than 1 year exhibited reduced static and dynamic spontaneous activity in the temporal and parietal lobes, including regions involved in basal ganglia–thalamus–cortex circuits, the DMN, and the ventral attentional network. Moreover, these abnormalities were associated with cognitive deficits and depression. These findings provide important clues to the pathomechanisms underlying hemodialysis -associated cognitive decline in patients with ESRD, as well as multiple potential neuroimaging markers for diagnosis, monitoring of disease progression, and treatment evaluation.

## Data Availability

The raw data supporting the conclusions of this article will be made available by the authors, without undue reservation.

## Footnotes

^1^ http://rfmri.org/dpabi

## Glossary

ESRD: End-stage renal disease
ESHD: End-stage renal disease patients undergoing maintenance hemodialysis
HC: Healthy control
ReHo: Regional homogeneity
sReHo: Static regional homogeneity
dReHo: Dynamic regional homogeneity
NCT-A: Digital connections test types A
NCT-B: Digital connections test types B
LTT: Line-tracing test
SDT: Serial dot test
DST: Digit symbol test
MoCA: Montreal cognitive assessment
MMSE: Mini-Mental State Examination
SDS: Self-rating Depression Scale
SAS: Self-rating Anxiety Scale
DMN: Default mode network
AN: Attention network
SMG: Supramarginal gyrus
ANG: Angular gyrus
PoCG: Postcentral gyrus
STG: Superior temporal gyrus
MTG: Middle temporal gyrus
CAU: Caudate nucleus
THA: Thalamus
MFG: Middle frontal gyrus
